# Community structure, diversity and function of endophytic and soil microorganisms in boreal forest

**DOI:** 10.3389/fmicb.2024.1410901

**Published:** 2024-10-02

**Authors:** Xi Luo, Guoyong Yan, Qinggui Wang, Yajuan Xing

**Affiliations:** ^1^School of Life Sciences, Qufu Normal University, Qufu, China; ^2^Library, Qufu Normal University, Qufu, China

**Keywords:** forest microbiome, ecosystem function, plant-associated microorganisms, plant endophytic microorganisms, boreal forests

## Abstract

**Introduction:**

Despite extensive studies on soil microbial community structure and functions, the significance of plant-associated microorganisms, especially endophytes, has been overlooked. To comprehensively anticipate future changes in forest ecosystem function under future climate change scenarios, it is imperative to gain a thorough understanding of the community structure, diversity, and function of both plant-associated microorganisms and soil microorganisms.

**Methods:**

In our study, we aimed to elucidate the structure, diversity, and function of leaf endophytes, root endophytes, rhizosphere, and soil microbial communities in boreal forest. The microbial structure and composition were determined by high-throughput sequencing. FAPROTAX and FUNGuild were used to analyze the microbial functional groups.

**Results:**

Our findings revealed significant differences in the community structure and diversity of fungi and bacteria across leaves, roots, rhizosphere, and soil. Notably, we observed that the endophytic fungal or bacterial communities associated with plants comprised many species distinct from those found in the soil microbial communities, challenging the assumption that most of endophytic fungal or bacterial species in plants originate from the soil. Furthermore, our results indicated noteworthy differences in the composition functional groups of bacteria or fungi in leaf endophytes, root endophytes, rhizosphere, and soil, suggesting distinct roles played by microbial communities in plants and soil.

**Discussion:**

These findings underscore the importance of recognizing the diverse functions performed by microbial communities in both plant and soil environments. In conclusion, our study emphasizes the necessity of a comprehensive understanding of the structure and function microbial communities in both plants and soil for assessing the functions of boreal forest ecosystems.

## Introduction

Many studies have demonstrated the crucial role of the soil microbiome in maintaining the health of terrestrial ecosystems (Chaparro et al., 2012; Jiao et al., 2018; Dubey et al., 2019; Banerjee and van der Heijden, 2023). This diverse community of bacteria and fungi contributes to various essential functions. One of its primary roles is nutrient cycles (Bahram et al., 2018; Jiao et al., 2018; Sokol et al., 2022), where microorganisms decompose organic matter, releasing essential nutrients such as nitrogen, phosphorus, and potassium for plant uptake. Additionally, specific fungi can form symbiotic relationships with plant roots, aiding in nutrient absorption and enhancing plant growth (Bonfante and Genre, 2010; Begum et al., 2019; De Mandal et al., 2021). Furthermore, the soil microbiome acts as a key player in disease suppression, with certain microorganisms inhibiting the growth of harmful pathogens (De Corato, 2020; Pascale et al., 2020). Soil microorganisms also contribute to soil structure and stability, playing a crucial role in preventing erosion and promoting water retention (Hartmann and Six, 2023). In essence, the soil microbiome is indispensable for sustaining healthy ecosystems and mitigating environmental challenges.

In forest ecosystems, plants provide a diverse array of niches for the growth and proliferation of microorganisms. The plant microbiota, consisting of bacteria, fungi, and so on, colonizes all accessible plant tissues (Trivedi et al., 2020). These microorganisms establish complex associations with plants, playing crucial roles in enhancing plant productivity and maintain health in natural environments (Laforest-Lapointe et al., 2017; Trivedi et al., 2020). Previous studies have highlighted the significance of root microbiota in promoting plant growth and resilience to various stresses (Philippot et al., 2013; Trivedi et al., 2020). Additionally, leaf-associated microorganisms influence host fitness, growth, resilience to abiotic stresses, and resistance to pathogens (Vorholt, 2012; Laforest-Lapointe et al., 2017; Perreault and Laforest-Lapointe, 2022). Positive correlations between the diversity of tree-associated microbiota and ecosystem productivity have been observed, while decreases in diversity are linked to disease states and propagation (Laforest-Lapointe et al., 2017; Perreault and Laforest-Lapointe, 2022). Moreover, a growing body of evidence suggests that diverse microbial communities associated with roots, leaves, and soil collectively contribute to enhancing plant fitness under environmental changes (Giauque et al., 2019; Hawkes et al., 2020; Angulo et al., 2022). For example, the rhizosphere microbiome can also form a biological protective barrier, reducing the invasion of pathogenic microorganisms, which is especially important when plants face environmental stresses such as drought or disease outbreaks (Mendes et al., 2013; Singh et al., 2023). Despite recognizing the vital functions of soil microorganisms and plant endophytes, previous studies predominantly focused on soil microorganisms and neglected the interconnected dynamics of soil, root, and leaf microorganisms, particularly in boreal forests (Hassani et al., 2018; Hawkes et al., 2020; Angulo et al., 2022). Importantly, there is a gap in our understanding of microbial endophyte community structure, diversity, and functions in boreal forests. Addressing this gap is crucial, as it could limit our comprehensive understanding of the functions of soil microorganisms and plant endophytes across ecosystems.

To comprehensively boreal forest ecosystem function, exploring the structure, diversity, and functional groups of microbial communities within leaves, roots, rhizosphere, and soil is essential. The microbial communities (including their structure, diversity, and functional groups) and their effects on plant systems can influence plant growth, health, and stress resistance, making it a critical aspect of understanding ecosystem dynamics. The living environment acts as a major selective force, shaping the composition of both soil microorganisms and plant endophytes, as highlighted by Trivedi et al. (2020). Thus, we hypothesize significant differences in microbial community structure and diversity across various sampling positions (including bulk soil (non-rhizosphere soil), rhizosphere soil, leaf and root). Soil microorganisms, with their primary function in promoting soil nutrient cycling, are complemented by rhizosphere microorganisms, which enhance the efficiency of plant nutrient absorption. Simultaneously, plant endophytic microorganisms contribute to the adaptability of plants to their environment. Consequently, we further hypothesize significant differences in the functional groups of microbial communities in different positions, meeting diverse plant needs and ecosystem functions. To investigate these hypotheses, *Larix gmelinii*, the dominant tree species in the boreal forest, was chosen. We collected samples, including leaves, roots, rhizosphere, and soil, from 36 *Larix gmelinii* trees. Employing high-throughput sequencing, we determined the microbial community structure in different positions, with a specific focus on endophytic microbial communities identified in leaves and roots.

## Materials and methods

### Site description

Our sampling was conducted in the boreal forest within Nanwenghe National Natural Reserve, located in the Greater Khingan Mountains, China (51°05′-51°39′N, 125°07′-125°50′E). The study area features a cold temperate continental climate characterized by a protracted cold winter (October–April) and a brief, warm summer (May–September). With a mean annual temperature of −2.4°C, monthly variations range from −26.3°C in January to 18.6°C in July. Annual precipitation averages 489.2 mm, with the majority falling in July and August. The soil in this region is classified as sandy (including many stones) based on an international soil classification system,^1^ and the mean soil thickness is 20 cm. Dominating the ecosystem is the *Larix gmelinii* tree species.

### Field sampling

In July 2021, we randomly selected 36 robust adult larch trees within the protected area for sampling. Each selected tree contributed comprehensive samples, including leaves, roots, rhizosphere, and bulk soil. To ensure representation, a minimum of ten fresh, intact, and fully expanded leaf samples were collected from the highest part of the tree crown, with sampling conducted from four directions. To capture the complete root system, spanning orders 1–5, excavation was carried out in four directions around the tree base. The collected leaf or root samples from each individual were then mixed and homogenized. Rhizosphere soil was obtained using the shaking-off method, where soil tightly attached to the root surface was designated as rhizosphere soil (Phillips et al., 2011). Bulk soil samples were gathered 2–3 meters away from the tree trunk in four directions. To minimize the influence of surface microbes, leaves and roots underwent prompt surface sterilization within 12 h of collection. This process involved sequential rinsing in deionized water, 95% ethanol, 0.5% sodium hypochlorite (NaOCl), and 70% ethanol, following established protocols (Pan et al., 2023). The rhizosphere soil or bulk soil from each tree was thoroughly mixed and sieved through a 2-mm mesh to eliminate plants, small animals, roots, and gravel. This process yielded four distinct samples from each tree: leaf, root, rhizosphere soil, and bulk soil. All samples were promptly flash-frozen in liquid diatomic nitrogen (N_2_) for transportation to the laboratory and subsequently stored at −80°C for nucleic acid extraction.

Part of the soil sample was used to determine soil properties. The soil pH value was measured i using a pH meter (SX7150, China). Ammonium nitrogen (NH_4_^+^) and nitrate nitrogen (NO_3_^−^) were measured using a continuous flow analyzer (Skalarsan+, Netherlands). The soil total organic carbon (TOC) and total N (TN) concentrations were measured using an automated TC/TN analyzer (multi N/C 3100, Analytik Jena AG, Germany). Soil total phosphorus (TP) was measured via spectrophotometer with molybdenum–antimony antibody colorimetry.

### DNA extraction

Total microbial DNA was extracted from pooled leaf and root tissue samples using Qiagen Plant DNeasy kits. For soil genomic DNA extraction in each sample, the MoBio PowerSoil DNA extraction kit (MO BIO Laboratories, Inc., Carlsbad, CA, USA) was employed following the manufacturer’s protocol, with minor adjustments for enhanced yield and purity. A uniform one-step PCR amplification process was applied to all samples, achieving normalization through primers designed to attach a 12-base-pair barcode and Illumina adaptor sequence during PCR. Targeting the 16S rRNA gene for bacteria and the ITS region for fungi amplicons, in alignment with established protocols, each sample underwent a single 25 μL PCR reaction. The resulting amplicons underwent processing using an Invitrogen Sequalprep PCR Cleanup and Normalization Kit. Following equal concentration pooling, the samples underwent sequencing using Illumina Hiseq 2500, generating pair-end 250-base pair reads. To ensure data accuracy, rigorous sequencing quality control measures were implemented to filter out low-quality reads.

### Bioinformatic analysis

The raw sequence data underwent processing using QIIME2 pipelines (Bolyen et al., 2019). This comprehensive procedure involved merging paired-end sequences into a single sequence of approximately 350 bp in length. To ensure data quality, sequences with a mean quality score less than 30 or with any series of five bases having a quality score below 30 were eliminated. Subsequently, the sequences were de-multiplexed into respective samples. Quality-controlled reads were subjected to denoising using the DADA2 plugin (Callahan et al., 2016), resulting in the identification of amplicon sequence variants (ASVs) with a 97% similarity. Taxonomy assignment for each ASV was achieved through a Naive Bayes classifier trained with eukaryotes ITS sequences from the UNITE database (version 7.2, Nilsson et al., 2019) and prokaryotic 16S sequences from the Greengenes database (version 13.5, DeSantis et al., 2006). The final ASV table was meticulously generated by excluding nonfungi or nonbacterial ASVs and potential false-positive ASVs with a low abundance of fewer than ten reads across all samples. To estimate alpha-diversity indices for bacteria and fungi, MOTHUR software (version 1.30.1) was employed. We employed FAPROTAX (Louca et al., 2016) and FUNGuild (Nguyen et al., 2016) to analyze the functional groups of bacteria and fungi in the soil. For bacterial Operational Taxonomic Units (OTUs), we compared them with the dataset obtained by FAPROTAX (script version 1.1), and the output functional table used the default settings. FUNGuild v1.0 was utilized to determine the functional groups of fungi, and it is a flat database hosted on GitHub.^2^ To prevent over-interpretation of fungal functional groups, we excluded the confidence level of “possible” and retained only the two levels of “highly probable” and “probable.” Communities that could not be identified or were identified as having multiple complex nutrition methods were unified as “undefined.”

### Statistical analysis

For statistical analysis, the Kolmogorov–Smirnov test was employed for a normality test, and Levene’s test was used for the homogeneity of variance test. We utilized the linear model with Tukey’s multiple comparison to evaluate the differences in microbial diversity indices, relative abundance of microphyla, and microbial functional groups among different sampling positions. Visualization of microbial communities and functional structures was conducted through nonmetric multidimensional scaling (NMDS) based on the Bray-Curtis distance. Additionally, the “ADONIS” function of the vegan package in R (999 permutations) was used to test the significance of the separation between successional stages. ANOSIM analysis was employed to detect differences within and between groups. All analyses were conducted in R (version 4.1.1, R Core Team, 2021).

## Results

### Microbial diversity

**Figure 1:**
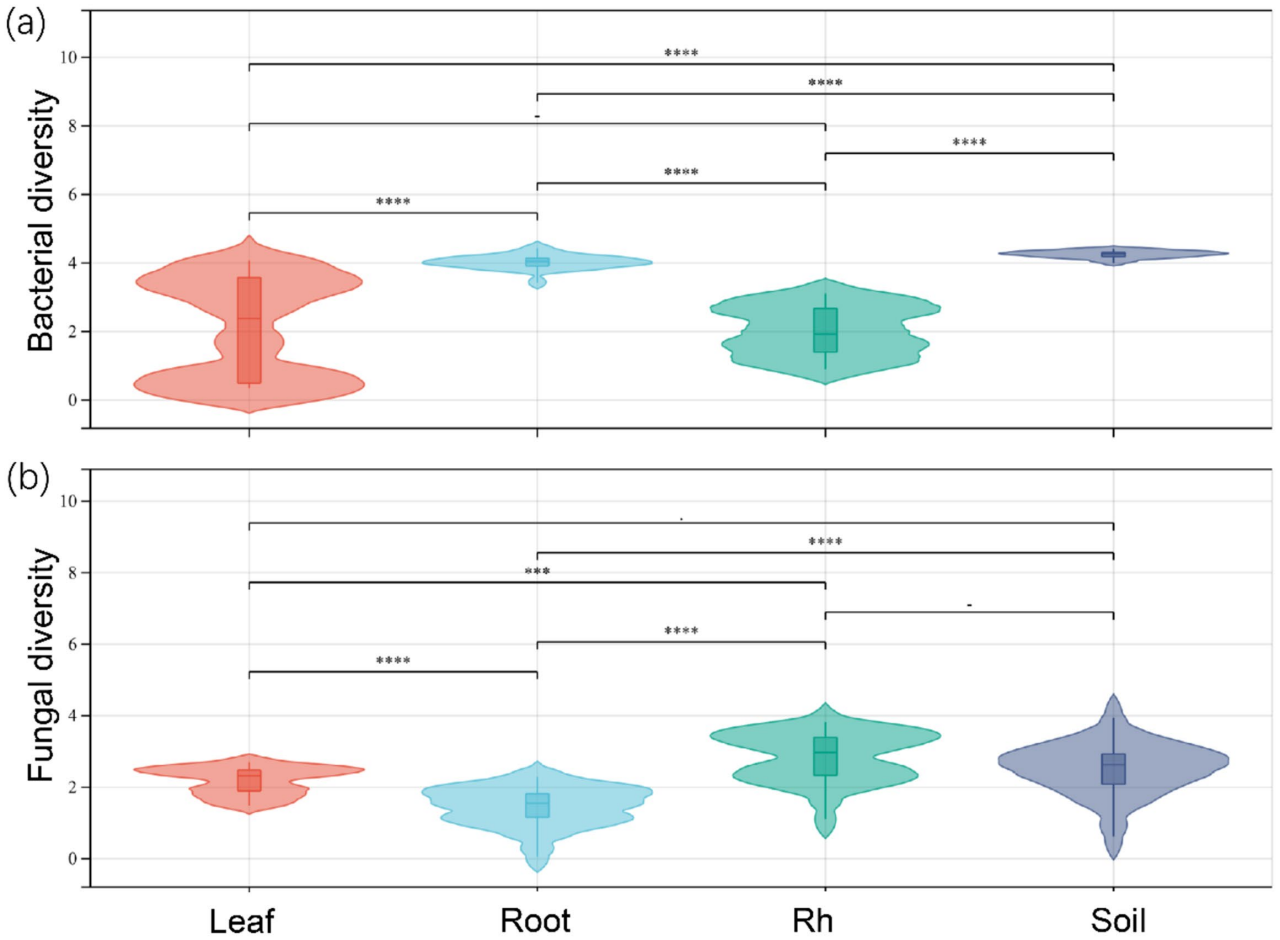
Bacterial diversity (a) and fungal diversity (b) of endophytic and soil microorganisms. Leaf, leaf endophyte; Root, root endophyte; Rh, rhizospheric microorganism; Soil, soil microorganism. One-way analysis of variance was used to compare differences in the microbial diversity of different positions. ****p < 0.0001; ***p < 0.001; **p < 0.01; *p < 0.05.

The soil properties in the study area are presented in Table 1. Significant differences in bacterial and fungal diversity, as assessed by the Shannon diversity index, were observed across leaf endosphere, root endosphere, rhizosphere, and bulk soil (Figure 1). However, distinctive patterns emerged when comparing bacterial and fungal diversity across different sampling positions. Bacterial diversity exhibited the highest values in bulk soil and the lowest in the leaf endosphere. In contrast, fungal diversity showed a different trend, with the highest values in the rhizosphere and the lowest in the root endosphere (Figure 1). In summary, the order of bacterial diversity was as follows: bulk soil (4.25) > root endosphere (4.02) > rhizosphere (2.17) > leaf endosphere (2.10). Similarly, the order of fungal diversity was rhizosphere (2.79) > bulk soil (2.68) > leaf endosphere (2.20) > root endosphere (1.73) (Figure 1).

**Table 1:** Soil properties in the study area.

Soil properties	pH	NH_4_^+^(mg·kg^−1^)	NO_3_^−^(mg·kg^−1^)	TN(g·kg^−1^)	TP(g·kg^−1^)	SOC(g·kg^−1^)
Study area	4.56 ± 0.15	25.68 ± 1.72	14.97 ± 4.29	1.31 ± 0.10	0.38 ± 0.02	23.66 ± 1.43

### Microbial community structure

**Figure 2:**
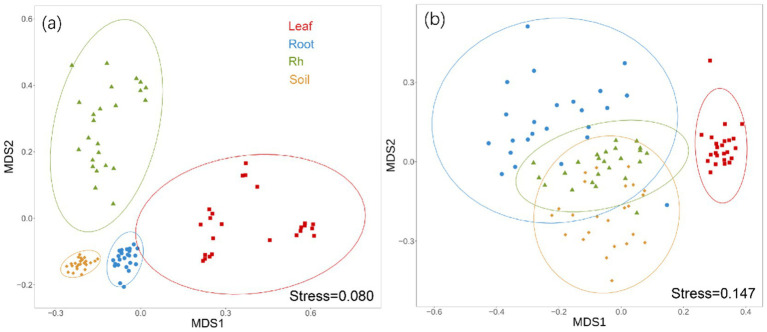
Non-metric multidimensional scaling analysis (NMDS) of bacterial community (a) and fungal communities (b) based on Bray-Curtis distances among different positions. Ellipses represent confidence intervals at 95%. The difference of bacterial and fungal community composition among different samples were tested by Adonis analysis based on Bray-Curtis distances. The PERMANOVA analysis showed that there were significant differences in bacterial and fungal communities between any two samples. Leaf, leaf endophyte; Root, root endophyte; RootR, rhizospheric microorganism; Soil, soil microorganism.

**Figure 3:**
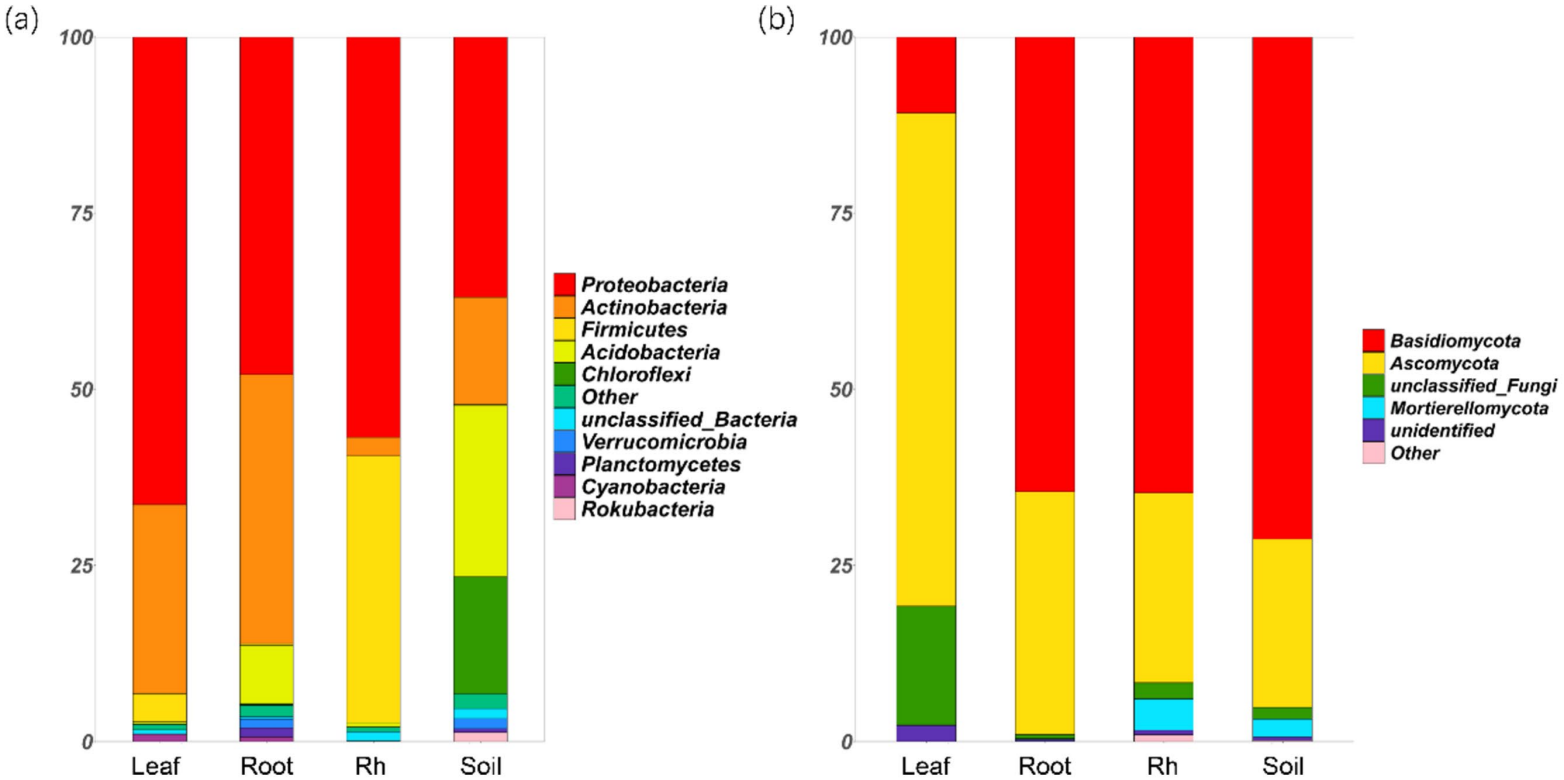
Relative abundances of the soil bacterial (a) and fungal (b) phyla in different positions. The phyla that relative abundances less than 1% are replaced with the other. Leaf, leaf endophyte; Root, root endophyte; RootR, rhizospheric microorganism; Soil, soil microorganism.

**Figure 4:**
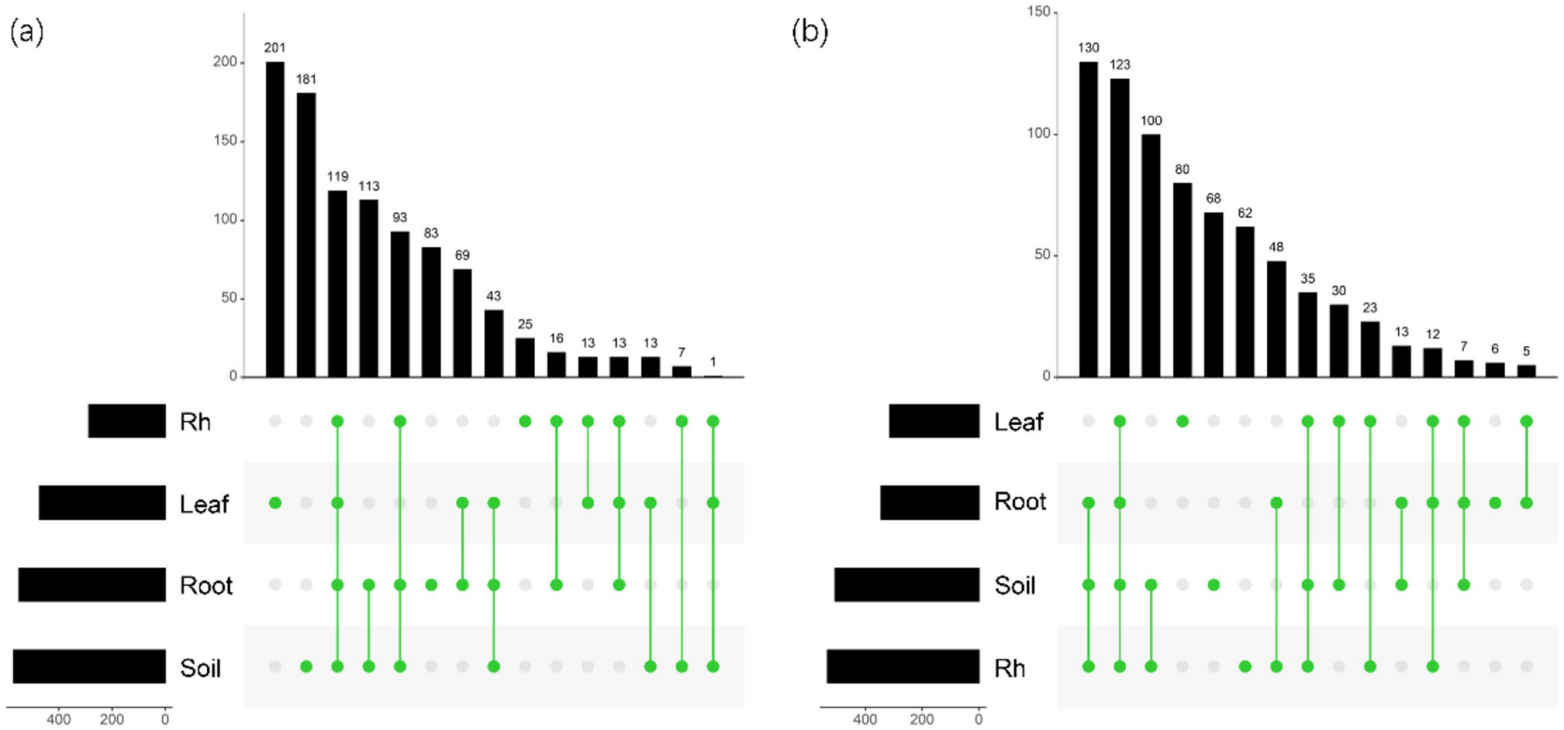
UpSet showing the number of species unique or shared between different positions. (a) The horizontal bar chart on the left shows the total number of bacterial species in each group; The bar chart above shows the number of species of different samples in different positions; The green dots in the middle indicate the number of species in each sample during each position. (b) The horizontal bar chart on the left shows the total number of fungal species in each group; The bar chart above shows the number of species of different samples in different positions; The green dots in the middle indicate the number of species in each sample during each position. Leaf, leaf endophyte; Root, root endophyte; RootR, rhizospheric microorganism; Soil, soil microorganism.

Bacterial and fungal community structures also show notable differences across leaf endosphere, root endosphere, rhizosphere, and bulk soil (Table 2; Figure 2). This variation in community structure may arise from differences in species composition (Figures 3, 4). The phyllosphere community primarily consists of bacteria from the phyla Proteobacteria, Actinomycetes, Firmicutes, Acidobacteria, and Chloroflexi, with members of phyla Proteobacteria constituting approximately 50% of the community composition in leaf endosphere, root endosphere, rhizosphere, and bulk soil (Figure 3). Plant endophytic communities are significant higher in Actinobacteria (approximately twofold or higher in relative abundance in the endophytic community than in the rhizosphere or bulk soil), while significant lower in Firmicutes (more than twofold lower in relative abundance in the endophytic community than in the rhizosphere), Acidobacteria, and Chloroflexi (more than twofold lower in relative abundance in the endophytic community than in the bulk soil). The diverse range of fungi colonizing both leaf and root tissues predominantly belong to the phyla Ascomycota and Basidiomycota (Figure 3). The rhizosphere and bulk soil are enriched in the phyla Mortierellomycota, while being depleted in plant endophytic communities (Figure 3). Among the unique species, bacterial and fungal species in the leaf endosphere were the most abundant compared to other tissues, while the rhizosphere had relatively fewer (Figure 4). This difference may primarily result from the distinct sources of endophytic microorganisms in leaves and roots.

**Table 2:** Differences on fungal and bacterial community composition were tested by adonis analysis based on Bray-Curtis distances.

Types	Groups	*R* ^2^	*p* value
Bacteria	Soil/Leaf	0.975	<0.001
	Soil/Root	0.983	<0.001
	Soil/Rh	0.908	<0.001
	Leaf/Root	0.674	<0.001
	Leaf/Rh	0.980	<0.001
	Root/h	0.823	<0.001
Fungi	Soil/Leaf	0.855	<0.001
	Soil/Root	0.312	<0.001
	Soil/h	0.256	<0.001
	Leaf/Root	0.717	<0.001
	Leaf/h	0.846	<0.001
	Root/h	0.138	<0.001

Leaf, leaf endophyte; Root, root endophyte; Rh, rhizospheric microorganism; Soil, soil microorganism.

### Microbial fungal groups

**Figure 5:**
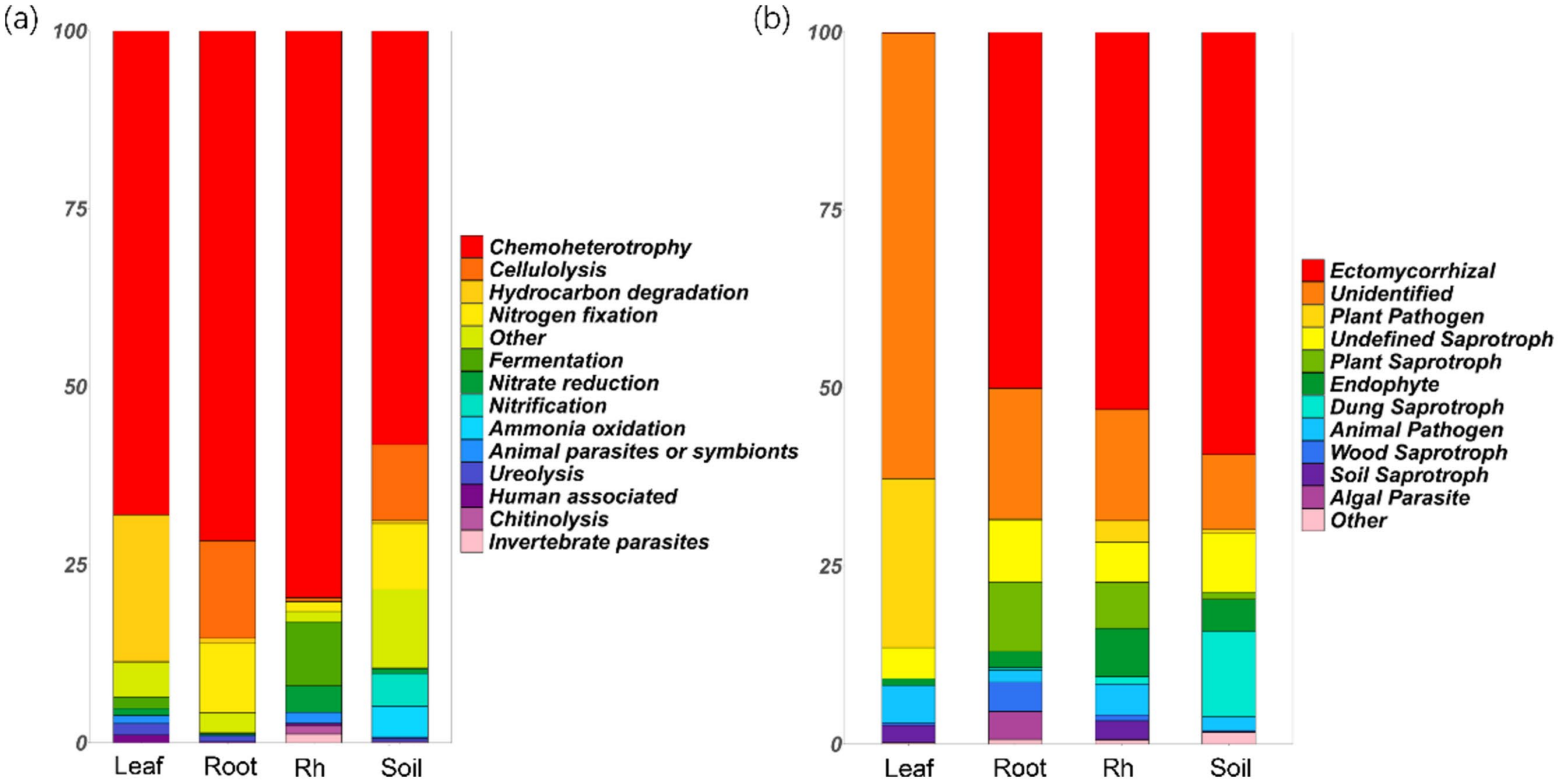
Relative abundances of bacterial functional groups (a) and fungal functional groups (b) in different positions. The groups with relative abundances less than 1% are grouped into “other.” Leaf, leaf endophyte; Root, root endophyte; RootR, rhizospheric microorganism; Soil, soil microorganism.

Chemoheterotrophic bacteria emerged as the most dominant functional group, with their relative abundance exceeding 50% across various ecological niches, including the leaf endosphere, root endosphere, rhizosphere, and bulk soil (Figure 5a). Notably, the leaf endosphere exhibited a significantly higher relative abundance of hydrocarbon-degrading bacteria compared to other positions (*p* < 0.05, Figure 5a). Additionally, the relative abundance of nitrogen-fixing bacteria was higher in root endosphere and bulk soil compared to the leaf endosphere and rhizosphere (Figure 5a). The rhizosphere showed the highest relative abundance of fermentation and nitrate-reducing bacteria, while bulk soil had the greatest relative abundance of ammonia-oxidizing and nitrifying bacteria (Figure 5a).

The relative abundance of ectomycorrhizal fungi reached its peak in the root endosphere, rhizosphere, and bulk soil, but notably, it was not as pronounced in the leaf endosphere (Figure 5b). Conversely, the relative abundance of plant pathogenic fungi was highest in the leaves, followed by the rhizosphere, and was least in the root endosphere (Figure 5b). Additionally, the relative abundance of saprophytic fungi was significantly higher in the root endosphere, rhizosphere, and bulk soil compared to the leaf endosphere (Figure 5b). Noteworthy is the observation that a majority of leaf endophytes could not be assigned to the three familiar functional groups (symbiotic, pathogenic, and saprophytic fungi), suggesting that their function may significantly differ from those in other positions (Figure 5b).

## Discussion

In this study, the diversity of bacteria and fungi in soil was significantly higher than that of endophytic bacteria and fungi, indicating that host could act as a filter, selecting specific populations (Trivedi et al., 2020). The higher bacterial diversity in bulk soil may be attributed to the diverse nutrient sources and environmental conditions available in this habitat (Bach et al., 2018; Xia et al., 2020). On the other hand, the lower bacterial diversity in the leaf endosphere could be influenced by factors such as limited nutrient availability or selective pressures exerted by the plant host (Bulgarelli et al., 2013; Trivedi et al., 2020). Similarly, the variation in fungal diversity across sampling positions underscores the importance of niche-specific interactions and ecological dynamics in the rhizosphere and endosphere compartments (Qian et al., 2019; Yang et al., 2021). The greater fungal diversity in the rhizosphere compared to bulk soil may be linked to the rhizosphere effect, where root exudates influence the structure of the microbial community (Haichar et al., 2008; Peiffer et al., 2013; Gong et al., 2023). Conversely, the lower fungal diversity in the root endosphere suggests potential selective processes or competition among fungal taxa for colonization within this niche (Qian et al., 2019; Wang et al., 2023). Overall, these results shed light on the complex interplay between plants and their associated microbial communities.

The significant variations in microbial diversity are accompanied by marked differences in bacterial and fungal community structures across the leaf endosphere, root endosphere, rhizosphere, and bulk soil. This divergence in community structure likely arises from difference in species composition. In the leaf endosphere, bacterial communities are predominantly represented by phyla Proteobacteria, Actinomycetes, Firmicutes, Acidobacteria, and Chloroflexi, with Proteobacteria comprising approximately 50% of the community composition across all sampled environments, which is consistent with findings from previous studies (Hardoim et al., 2008; Compant et al., 2021). Moreover, plant endophytic communities exhibit enrichment in Actinobacteria, while displaying depletion in Firmicutes, Acidobacteria, and Chloroflexi compared to the rhizosphere and bulk soil. The observation is consistent with previous study (Liu et al., 2017). The fungal communities inhabiting both leaf and root tissues demonstrate extensive diversity, primarily composed of phyla Ascomycota and Basidiomycota (Zuo et al., 2021). Furthermore, Mortierellomycota phyla were enriched in the rhizosphere and bulk soil, while was depleted in plant endophytic communities. The differences in fungal community composition between plant tissues and bulk soil suggest selective colonization processes within the plant ecosystem (Trivedi et al., 2020; Guo et al., 2021). Additionally, the analysis of specific species reveals that bacterial and fungal species in the leaf endosphere exhibit the highest endemism compared to other tissues, with relatively fewer specific species in the rhizosphere. This observation may be attributed to the distinct sources of endophytic microorganisms in leaves and roots, highlighting the influence of plant host specificity and environmental factors on microbial community assembly (Vandenkoornhuyse et al., 2015; Xiong et al., 2021). These results underscore the intricate interplay between plant-associated microbial communities and their respective habitats, emphasizing the importance of considering both microbial diversity and community structure in understanding the ecological dynamics within the plant microbiome. This study provides insights that enhance our understanding of the complex relationships between plants and their associated microbial communities, highlighting the need for further investigation into the mechanisms driving microbial community assembly and function within different plant organs (Bulgarelli et al., 2013).

The difference of microbial community structure and diversity at different positions may lead to the difference in microbial community function. Our study highlights the diverse functional roles played by microbial communities across various ecological niches within the plant ecosystem. Chemoheterotrophic bacteria were the dominant functional group, with a relative abundance exceeding 50% across all sampled positions. Interestingly, significantly higher relative abundance of hydrocarbon-degrading bacteria (includes species like *Pseudomonas aeruginosa*, *Rhodococcus erythropolis*, *Mycobacterium vanbaalenii*, *Sphingomonas paucimobilis*, *Pseudomonas putida*, and so on) was found in leaf endosphere compared to in other positions, suggesting a potential role in detoxification or metabolism of organic compounds in leaf endosphere (Gupta et al., 2017; Feng et al., 2017). Moreover, distinct patterns in specific bacterial functional groups were also observed across different sampling positions. The root endosphere and bulk soil showed a higher relative abundance of nitrogen-fixing bacteria compared to the leaf endosphere and rhizosphere, highlighting their importance in nitrogen cycling and plant nutrition (Beltran-Garcia et al., 2021; Zhang et al., 2022). Conversely, the rhizosphere displayed a higher relative abundance of fermentation bacteria and nitrate-reducing bacteria, while the bulk soil harbored a greater prevalence of ammonia-oxidizing bacteria and nitrification bacteria, indicating niche-specific microbial metabolic activities (Singh et al., 2022). In terms of fungal functional groups, ectomycorrhizal fungi reached their peak relative abundance in the root endosphere, rhizosphere, and bulk soil, underscoring their symbiotic association with plant roots and potential roles in nutrient uptake (Philippot et al., 2013). Conversely, plant pathogenic fungi exhibited their highest relative abundance in leaves, suggesting a potential threat to plant health within this microenvironment (Eberl et al., 2020; Trivedi et al., 2020). Additionally, the root endosphere, rhizosphere, and bulk soil displayed a significantly higher relative abundance of saprophytic fungi compared to the leaf endosphere, highlighting their role in organic matter decomposition and nutrient cycling in soil-associated habitats (Schappe et al., 2020). Notably, a majority of leaf endophytes could not be assigned to familiar functional groups, suggesting unique functional roles that may significantly differ from those observed in other positions (Trivedi et al., 2020). This underscores the need for further exploration to elucidate the specific functions and ecological significance of these enigmatic leaf endophytes within the plant microbiome. Overall, these findings provide valuable insights into the functional diversity and ecological roles of microbial communities in boreal forests, contributing to our understanding of microbial-mediated processes essential for plant health and ecosystem functioning (Berendsen et al., 2012).

## Conclusion

In conclusion, our study elucidates the intricate relationships between microbial diversity, community structure, and functional roles in a boreal forest. The results underscore the significance of considering both bacterial and fungal communities in understanding the dynamic interplay between plants and their associated microbiota. Chemoheterotrophic bacteria emerged as the predominant functional group, exhibiting diverse metabolic capabilities across sampled positions, while fungal functional groups exhibited distinct distribution patterns that reflected their ecological roles. Notably, the leaf endosphere harbored unique microbial communities with specialized functional attributes, suggesting niche-specific adaptations and potential contributions to plant health and fitness. Furthermore, differences in specific bacterial functional groups, such as nitrogen-fixing bacteria and ammonia-oxidizing bacteria, across the sampling positions emphasize the significance of microbially mediated nutrient cycling and plant-microbe interactions. Future research should be focused on unraveling the functional significance of microbial communities in mediating plant-microbe interactions and ecosystem processes, ultimately enhancing our ability to harness the beneficial contributions of microbes for sustainable agriculture and environmental stewardship.

## Data Availability

The datasets presented in this study can be found in online repositories. The names of the repository/repositories and accession number(s) can be found at: https://www.ncbi.nlm.nih.gov/, SUB14194707.

## Footnotes

^1^http://www.iuss.org/index.php?article_id=175

^2^https://github.com/UMNFuN/FUNGuild
